# Allegheny County Medical Society

**Published:** 1889-08

**Authors:** 


### Society Reports.

### ALLEGHENY COUNTY MEDICAL SOCIETY.

Special Meeting, May 21st, 1889.

W. F. Knox, M. D., President, in the chair.

Dr. Buchanan read a paper entitled

### TWO INCOMPLETE OVARIOTOMIES; ONE FATAL.

Dr. McCann reported,

### TWO CASES OF NEPHRECTOMY.

I operated upon a case of movable kidney. The kidney could be readily detected in the abdomen above the umbilicus, could be pushed beyond the median line and downward to the anterior superior spinous process of the ilium. The woman was emaciated and weak; had suffered constant pain, sickness at the stomach, and abdominal tenderness. The kidney was easily reached by the lumbar incision, and pressure upon the abdominal wall in front brought the kidney into its normal position. The peri-renal fat was divided and the true fibrous capsule exposed. The ease with which the kidney could be grasped and held was remarkable, and demonstrated the advantage of the lumbar operation for removal of the kidney, when not enlarged beyond double its normal size. After pushing the tumor down to its normal position, freeing it from peri-renal fat, four strong silk-worm gut sutures were passed so as to include the capsule and cortical structure of the kidney, the lumbar fascia and a portion of the deeper muscles. The kidney was held in its normal position, and the ligatures were drawn tightly and tied; their ends being cut short and left in the wound, and the kidney thus securely "anchored"

in its natural location. A drainage tube was placed in the wound, the entire wound united by silver sutures passed deeply through the muscles, and an antiseptic dressing applied. No shock followed the operation, and there was no rise of temperature, except on one occasion when it reached 101° F. No renal disturbance whatever followed. The wound united kindly; the drainage tube was removed on the seventh day, and thus far the patient has progressed very favorably. A pad is worn over the abdomen to support the kidney.

The second case was one of supposed cancer of the kidney. A tumor existed in the right loin, extending almost to the umbilicus and down to a little below the anterior superior spinous process of the ilium. It was very painful and had been noticed first eight months ago. A careful microscropical examination of the urine, which was loaded with pus, apparently revealed the presence of cancer cells, and the case was supposed to be one of cancer of the kidney. There was an obscure sense of fluctuation in the tumor, and on exploring with a hypodermic syringe a quantity of pus was withdrawn. The patient was etherized, and an incision through the muscles in the loin exposed the surface of the kidney. The incision was deepened in the direction of the fluctuation through a portion of normal kidney structure. About a pint and a half of thick, not unhealthy looking, pus was discharged. The wound in the kidney was enlarged and the pelvis and calyces explored in every direction, in the hope of being able to detect a calculus, but none was discovered. A large drainage tube was placed in the wound and an antiseptic dressing applied. During the first forty-eight hours there was a copious discharge of pus, but this has gradually diminished. Two weeks have now passed since the operation; the woman is able to sit up, to walk, and is free from pain; the enlargement has almost disappeared and her health is steadily improving.

DR. McMULLEN: I have had in my practice, since March 10, eight cases of

CEREBRO-SPINAL MENINGITIS

of the epidemic variety. The whole number of cases have existed in five families and about five squares apart. Three cases have been fatal, one after five hours, one sixteen hours after first symptoms, and one twenty-seven days after the first development of symptoms. One has recovered with the loss of the sight of one eye, and I think there will be some permanent disability of the left leg. The treatment consisted of calomel and rhubarb for purgation, and of large doses of bromide and iodide of potash. For pain, opium and belladonna were administered.

DR. KEARNS: We had an epidemic of cerebro-spinal meningitis three years ago. At that time I had a number of cases. I became convinced that quinine was the great remedy. Symptoms are to be combated as they arise, but as to the general treatment, quinine is most efficacious. I have my doubts about its being an infectious disease.

DR. THOMAS: I have used chloral, I suppose, the last twelve years in cases of cerebro-spinal meningitis, and I approach these cases now with a great deal of assurance. It must be given in large doses; not in five grain doses, nor in forty grain doses, but in doses sufficient to control the symptoms; that is, the dose may be five grains; it may be twenty.

Dr. Stewart reported

### A CASE OF STRICTURE OF THE URETHRA.

I desire to report a case of stricture of the urethra, which has some interesting points. The patient came to me with a history of having contracted gonorrhœa four months previously, which soon became a gleet. His principal trouble, however, was a difficulty in beginning the act of micturition, associated with a difficulty in completing the act, owing to spasmodic contractions of the compressor urethræ muscles. During the past three months he had been subjected to a course of introduction of sounds without benefit. I examined his urethra with the urethrometer and found it to have a caliber of 40 F., but at the meatus there was a contraction to 34 F. I incised this stricture to 40 F., but did not introduce a sound, as at that time I had none sufficiently large to be of any service.

Three days afterward the patient returned and reported that from the time his meatus was cut, the trouble in urinating had ceased. The gleet was subsequently cured by the introduction for few times of a No. 38 and 40 F. sound. The interesting point in his case is the interference a stricture of the meatus capable of admitting a No. 34 sound may exert on the act of micturition, and the immediate relief obtained by removal of the stricture.

I have so often observed the beneficial effects obtained by cutting the meatus where it is contracted, that I make it a routine practice in the treatment of chronic urethral troubles, to cut the meatus if contracted, preliminary to the further treatment of the urethra. In cutting the meatus I do not follow the directions of the authorities, to cut the flaccid penis, but insert a bivalve meatoscope, and cut the meatus when rendered tense. By this means there is less pain and the incision is more likely to be vertical than if the tissues were not put on the stretch.

DR. THOMAS: We know that various conditions about the penile organ produce reflex troubles. Where a stricture of the urethra exists, and it becomes necessary to incise it, the rule is to afterwards pass full-sized sounds in order that the incision may be filled in with a plug of new tissue; if we do not do so, when the parts have healed, they remain as they were before the cutting. As the doctor did not follow this rule, but at the same time succeeded in curing his patient of his symptoms, I suspect that these were of reflex origin. The mere fact that the patient's previous attendant had passed a pretty full-sized sound, amounts to nothing. Every penis has its own standard as to the caliber of its urethra. Some time ago I treated a young man about twenty years of age, who required a No. 40 F. sound before his strictures were completely dilated.

I agree with the doctor that when the meatus, or stricture immediately back of it, needs cutting, it is correct to put the parts upon the stretch. I usually do this little operation with the Otis urethrotome, inverted. By thus putting them upon the stretch the incision is more precise, and less bleeding follows.

DR. LEMOYNE: Diseases of the urinary passages are characterized by such a great variety of demonstrations that unique cases are frequently encountered. It is a fact very generally admitted that simple incision of urethral stricture is almost invariably ineffective. On the contrary, it must be followed by repeated dilatations to prevent the primary union of the divided tissue, and secure the development of a cicatricial tract by granulation, with the effect of increasing the calibre of the canal at the contracted portion. With all due regard to the observations reported in this case I am disposed to attribute the irritation to a diseased filament of nerve which was divided by the incision.

